# Motivation, Discipline, and Academic Performance in Physical Education: A Holistic Approach From Achievement Goal and Self-Determination Theories

**DOI:** 10.3389/fpsyg.2020.01808

**Published:** 2020-07-31

**Authors:** Fernando Claver, Luis Manuel Martínez-Aranda, Manuel Conejero, Alexander Gil-Arias

**Affiliations:** ^1^Centre for Sport Studies, Rey Juan Carlos University, Fuenlabrada, Spain; ^2^Faculty of Sport, Catholic University of San Antonio (UCAM), Murcia, Spain; ^3^Neuroscience of Human Movement Research Group (Neuromove), UCAM, Murcia, Spain; ^4^Faculty of Sport Science, University of Extremadura, Cáceres, Spain

**Keywords:** teacher climate, motivation, structural equations model, discipline, academic performance, Physical Education

## Abstract

The analysis of disciplined behaviors and academic performance in a school context has become one of the main concerns within the educational community. Physical Education is highlighted as a key subject to analyze students’ behavior. Researchers and Physical Education teachers are interested on the motivational processes that predict positive student outcomes in order to support them. Thus, the main purpose was to determine a predictive model of disciplined behaviors and academic performance in Physical Education students. The Achievement Goal Theory and Self-Determination Theory acted as the theoretical framework. A total of 919 Spanish secondary school students participated in the study. The studied variables were task-oriented motivational climate, basic psychological needs, autonomous motivation, disciplined behavior, and academic performance. Data collection included Spanish validated questionnaires. The Mplus statistical program was used to perform a structural equation model of prediction. It included antecedents (task-oriented climate), motivational processes (basic psychological needs and autonomous motivation), and consequences (disciplined behavior, Physical Education and overall students’ performance). The results revealed that positive outcomes (discipline and academic performance in Physical Education) were positively predicted by autonomous motivation; autonomous motivation was positively predicted by basic psychological needs and these, in addition, by the task-oriented climate. The results highlighted the importance of the task-oriented motivational climate and the mediating role of the basic psychological needs and autonomous motivation in order to generate these positive student outcomes in Physical Education. This study could be a useful resource for teachers, since it offers the motivational variables that lead students to achieve disciplined behaviors and academic performance in Physical Education. Intervention programs based on the results of the present study could be applied in Physical Education classes in order to obtain better behavioral as well as cognitive positive student outcomes.

## Introduction

The understanding of cognitive mechanisms related to students’ discipline behaviors and academic performance has become the most worrisome aspect to secondary Physical Education (PE) teachers (Gutiérrez and López, 2012b). Thus, discipline in the school environment has become one of the main concerns of the educational community since it is a key indicator that the teaching–learning process is carried out successfully. The educational structure aims to promote prosocial behaviors in order to generate the most favorable conditions for the teaching–learning process that will allow the student to achieve an adequate performance (Barkoukis et al., 2014; Sánchez-Oliva et al., 2014).

Teaching strategies are essential to create an adequate classroom context that enables the implementation of planned tasks. In fact, teachers who promote a conducive environment for learning and engagement, in which students collaborate in its development, will achieve the learning objectives (Noltemeyer et al., 2019; Núñez and León, 2019). On the contrary, those teachers who do not encourage a classroom context with involved, autonomous, and participatory students will have more difficulties in achieving the planned teaching objectives (Granero-Gallegos et al., 2020a). Regarding the teacher’s contribution to the disciplined behaviors promotion, the proper application of teaching skills and attitudes reduces disciplinary problems. Teaching skills in PE allow a better group control and give the teacher more time for corrections and provide feedback to students, increasing their participation, autonomy, and effectiveness in the classroom, and consequently academic achievement (Gutiérrez et al., 2009; Wade et al., 2020).

The relationship between teacher skills and student academic performance has been extensively studied (Taylor et al., 2014). Participative methodologies that focus the teaching–learning process on the student, positive corrections, and giving autonomy to students are associated with positive consequences (Gil-Arias et al., 2020), such as disciplined behaviors (Gutiérrez et al., 2010) and academic performance in PE (Cid et al., 2019). However, the very complex reality of PE lessons in secondary education sometimes forces the teacher to stop focusing on the students’ academic performance to give priority to more controlled instructional approaches in order to avoid disruptive behaviors (Granero-Gallegos et al., 2020a).

Among the difficult conditions of the classroom context, student motivation is highlighted as a key variable when analyzing secondary school discipline behaviors and academic achievement (León et al., 2015; Rahimi and Karkami, 2015; Chik and Abdullah, 2018) but also in PE achievement (Gutiérrez and López, 2012b; Sevil et al., 2017). In this regard, research in the psychosocial field provides an appropriate framework to integrate learning skills with coexistence skills. Conflict, disruptive behavior, disobedience to teachers’ instructions, and non-compliance with sanctions are phenomena closely linked to school amotivation (Vera and Moreno-Murcia, 2016; Anderson et al., 2019). Thus, this research is based on the Achievement Goal Theory (AGT) (Nicholls, 1984) and the Self-Determination Theory (SDT) (Ryan and Deci, 2020) as the framework that allows to explain and predict how the PE students’ behavior can be regulated (Vasconcellos et al., 2019).

The interpretation that people are intentional organisms, directed by certain objectives and acting rationally in accordance with them, is the fundamental idea of the AGT (Nicholls, 1984). In achievement contexts, beliefs consistently guide behavior. This theory assumes that the greatest point of interest for individuals in performance contexts such as PE lies in demonstrating competence and ability. Thus, the motivational climate transmitted by the teacher through the structuring and teaching pathway of PE classes may be a task-oriented motivational climate, where skill judgment is based on the level of task mastery being achieved (striving to improve), or an ego-oriented motivational climate, where social comparison between students is promoted, understanding success when they show greater skill than others (Wallhead et al., 2013).

On the other hand, the SDT has been one of the theoretical models that have contributed the most to a better understanding of the cognitive, emotional, and behavioral patterns related to student progress (Ryan and Deci, 2020), especially in PE (Van Den Berghe et al., 2014; Vasconcellos et al., 2019). According to this theory, the origin of motivation can be more internal or external for the student (more or less self-determined) depending on if they are freely engage in their activities (Ryan and Deci, 2017). Specifically, SDT distinguishes between different dimensions of motivation (autonomous, controlled, and amotivation). Autonomous motivation is the most self-determined one and involves the behavior regulation with the experiences of volition, psychological freedom, and reflective self-endorsement (Aelterman et al., 2012). Studies developed in secondary school PE students have linked the more self-determined motivation to positive and adaptive outcomes at affective (e.g., well-being) (Standage et al., 2012), cognitive (e.g., academic achievement) (Ntoumanis and Standage, 2009), and behavioral levels (e.g., discipline) (Moreno-Murcia et al., 2011; Zimmerman and Kitsantas, 2014). Within the SDT, three basic psychological needs (BPNs) lead to self-determined behaviors (Ryan and Deci, 2017). The BPN of autonomy is satisfied when the students have the initiative in their behavior and the opportunity to choose; the BPN of competence is linked to the effective interaction with the environment, while the BPN of relatedness is related to positive interactions and group membership. Previous research has demonstrated the positive predictive capacity of BPNs on autonomous motivation in PE (Koka, 2014; Sánchez-Oliva et al., 2014; Ferriz et al., 2016; Van Den Berghe et al., 2016).

Complementary to the SDT, the Hierarchical Model of Motivation (Vallerand and Ratelle, 2002; Vallerand, 2007) was created in order to improve and relate the constructs of the SDT and originated the integral analysis of motivational and cognitive processes (McCarthy, 2011). The Hierarchical Model of Motivation explains the determinants of motivation processes and its consequences, becoming the main theory to explain motivation in the PE, sport, and exercise field (Clancy et al., 2016). In this way, the motivational climate that the teacher promotes in PE lessons constitutes one of the fundamental elements influencing the satisfaction of BPNs, acting as mediators between social factors and the self-determined type of regulation experienced by students (Tessier et al., 2010). According to previous research, SDT and AGT could be integrated in order to achieve a better motivational process understanding and its positive consequences (Duda, 2013). Furthermore, in a PE context, the teacher encourages a motivational climate, characterized by a variety of challenging tasks at the personal level, where cooperation is necessary to achieve a common goal, and where the students can have their own initiative, covering their BPNs. This will lead to a more self-determined motivation, having positive affective, cognitive, and behavioral consequences (Cox and Williams, 2008; Abós et al., 2017).

Previous research on the teaching of PE has been limited by the analysis of antecedents, motivational processes, and consequences in an isolated manner (Cid et al., 2019). Although the discipline has been a well-studied construct in the general educational context (Zimmerman and Kitsantas, 2014), studies in the field of PE are less abundant. Research has pointed out that the perception of a task-oriented classroom environment was positively related to self-determined reasons to maintain disciplined behavior. It was also associated with the student’s perception, mainly related to strategies based on responsibility and intrinsic reasons that the teacher used to maintain discipline (Spray and Wang, 2001; Gutiérrez and López, 2012a; Granero-Gallegos et al., 2020b). The study conducted by Spray and Wang (2001) in a group of English students reported that those who had a higher feeling of competence in PE expressed more self-determined reasons for maintaining and adequate behavior during the sessions. On the other hand, Moreno et al. (2011) analyzed a sample of Spanish secondary school students, determining that intrinsic motivation was positively related to the three BPNs, as well as with the discipline behavior. Other studies analyzed students’ discipline and self-regulation measures as a predictor of academic performance and did not only analyze the disciplined behavior as a consequence (Zimmerman and Kitsantas, 2014).

Academic performance at school, measured as the final score of the student (Sternberg, 2015), has been traditionally studied and explained by individual variables, mainly intellectual, but also by personality and contextual factors. Some authors state that the cognitive variables are the most important for predicting academic achievement and explaining most of the phenomena (Poropat, 2011). However, other authors support that the study of contextual and personality variables provides a better and more complete explanation of academic performance. In a recent study, Baños et al. (2020) presented a predictive model of academic performance based on the individual’s satisfaction. Student motivation is shown as a relevant variable because motivation is related to the learning goals that students have, which, in turn, evoke different mental situations in students, resulting in them having a positive or negative attitude toward study. This determines the effort invested to achieve learning and academic performance. A meta-analysis presented by Taylor et al. (2014) highlighted the role of motivation on school achievement. They also found that self-determined motivation was associated with higher academic performance. In PE, academic performance and metacognitive skills have been predicted by motivational variables in previous research (Chatzipanteli et al., 2015; Kirby et al., 2015; Sevil et al., 2017). In a longitudinal study, Barkoukis et al. (2014) indicated that a more self-determined motivation obtained higher academic performance in PE and, in addition, a less self-determined motivation explained lower qualifications.

Finally, several studies demonstrated the association between academic performance and disciplined behaviors in a high school context (Zimmerman and Kitsantas, 2014; Noltemeyer et al., 2019), but scientific research testing predictive models of both disciplined behavior and academic achievement in PE (Gutiérrez and López, 2012b) is limited. To our knowledge, these studies do not integrate AGT and SDT constructs. At this point, and given the relevance of motivational variables to regulate behavioral and cognitive outcomes of students in PE, the aim of this study was to determine a predictive model of disciplined behaviors and academic performance (overall and specific) in PE secondary school. We hypothesized that (1) task-oriented climate will predict autonomous motivation through the BPNs (Cox and Williams, 2008) and that (2) autonomous motivation will predict disciplined behaviors (Spray and Wang, 2001) and (3) academic performance (Cid et al., 2019).

## Materials and Methods

### Design and Participants

A cross-sectional study design with on-probability-based sampling was used. The sample consisted of 919 secondary school students, from 10 educational centers from two regions in Spain, aged between 12 and 18 years old (*M* = 14.63; *SD* = 1.54). The sample was composed of students from both genders, male (*n* = 433, *M*_age_ = 14.62, *SD*_age_ = 1.61) and female (*n* = 486, *M*_age_ = 14.64, *SD*_age_ = 1.49). In order to represent the different characteristics of the population, 42 classes (clusters) were randomly selected. Each cluster consisted of a group of approximately 21 students.

### Measurements

The Spanish version (Cervelló et al., 2010) of the Learning and Performance Orientation in PE Classes Questionnaire (Papaioannou, 1994) was used to measure task-oriented motivational climate in PE classes, as in previous research (Gil-Arias et al., 2020). The questionnaire begins with the sentence “In my PE classes…” and the factor is composed of 13 items (e.g., “The PE teacher is most satisfied when all the students learn something new” or “I am very satisfied when I learn new skills and games”). The factor reported a McDonald’s Omega of 0.86. Confirmatory factor analysis showed adequate adjustment indexes: χ^2^ = 749.90; *p* < 0.001; χ^2^/df = 2.44, CFI = 0.93, TLI = 0.92, RMSEA = 0.04, SRMR = 0.07.

The Spanish version (Moreno-Murcia et al., 2008b) of the BPNs in the Exercise Scale (Vlachopoulos and Michailidou, 2006) was applied to measure BPNs in the PE context, as in previous research (Jiménez et al., 2015). The questionnaire begins with the initial question “In my PE classes…” and it is composed of a total of 12 items, of which four measured autonomy (e.g., “The way I conduct classes is an expression of myself”), four related to competence (e.g., “I am able to manage the demands of a PE class”), and the other four measured the relatedness factor (e.g., “I interact in a very friendly way with the rest of the class”). Each factor reported a McDonald’s Omega of 0.81, 0.81, and 0.91, respectively. Confirmatory factor analysis showed adequate adjustment indexes: χ^2^ = 168.172; *p* < 0.001; χ^2^/df = 3.36, CFI = 0.96, TLI = 0.95, RMSEA = 0.05, SRMR 0.05.

The Spanish version (Moreno-Murcia et al., 2009) of the Perceived Locus of Causality Scale (Goudas et al., 1994) was used to measure autonomous motivation in PE, as in previous research (Ferriz et al., 2016). The questionnaire begins with the question “I participate in the PE classes…” and the factor is composed of eight items (e.g., “because PE is stimulating”). Autonomous motivation was calculated through intrinsic regulation (e.g., “because I enjoy learning new skills”) and identified regulation (e.g., “because I can learn skills that could be used in other areas of my life”) (Haerens et al., 2010). The factor reported a McDonald’s Omega of 0.97. Confirmatory factor analysis showed adequate adjustment indexes: χ^2^ = 452.908; *p* < 0.001; χ^2^/df = 2.96, CFI = 0.95, TLI = 0.94, RMSEA = 0.05, SRMR = 0.07.

The Spanish version (Cervelló et al., 2004) of the Discipline and Indiscipline Behavior in PE Inventory was used to measure discipline behaviors in PE classes, as in previous research (Gutiérrez and López, 2012b). The questionnaire begins with the statement “In my PE classes…” and the factor consists of 10 items (e.g., “you address yourself with respect to the teacher”). The factor reported a McDonald’s Omega value of 0.87. Confirmatory factor analysis showed adequate adjustment indexes: χ^2^ = 399.68; *p* < 0.001; χ^2^/df = 2.42, CFI = 0.99, TLI = 0.99, RMSEA = 0.04, SRMR = 0.04.

All the instruments were anchored on a Likert scale ranging from 1 (*strongly disagree*) to 5 (*strongly agree*).

*PE academic performance* and *Overall academic performace* were measured through a single item that reported the qualifications in the previous evaluation in PE and the rest of the subjects, respectively. This type of measure has been used in previous research (Baños et al., 2020).

### Procedures

The research was fully approved by the Ethics Committee of the local University following the guidelines of the Helsinki Declaration. In order to carry out the research, PE teachers and directors of the secondary schools were contacted. All students and their parents or legal tutors were informed about the study, which was anonymous. Subsequently, they signed a consent form to voluntarily participate in the study. Data collection occurred directly in the PE classes. An investigator provided questionnaires to the participants and informed them about how to fill them in, solving the questions that might appear during the process, ensuring an adequate concentration climate and avoiding the presence of the PE teacher. The estimated time to complete the instruments was 15 min.

### Statistical Analysis

Data analysis was performed using the statistical programs IBM SPSS Statistics 25.0 (IBM Corp, 2017) and MPlus 7.4 (Muthén and Muthén, 2012). The psychometric properties of the questionnaires were calculated, including an initial exploratory factor analysis (EFA) and confirmatory factor analysis (CFA) to test the instruments’ factor structure. The reliability of the study measurements was analyzed through McDonald’s Omega because Cronbach’s Alpha requires equal loads for all items in the same factor (Zhang and Yuan, 2016) and also that the numerical data should be continuous. Moreover, McDonald’s Omega has shown evidence of better accuracy to Likert-type ranging responses and values above 0.70–0.80 implying reliable measures (Revelle and Zinbarg, 2009).

The structural equation model (SEM) proposed was analyzed with the aim of testing the association between the study variables. The indicators of the latent variables in the SEM were the items of the different scales. To estimate the value of the parameters and the adjustment indexes in both CFA and SEM, robust maximum likelihood (MLR) estimation method was employed due to the Likert nature of the items. Model adjustment was assessed with a combination of the χ^2^/df test (<5) and the adjustment indexes (Kline, 2011). The *P*-value was established at level 0.05. A comparative adjustment index (CFI) and Tucker–Lewis index (TLI) close to or above 0.90 together with a root mean square error of approximation value close to or below 0.06 and the standard root mean square residual (SRMR) close to or below 0.08, respectively, were considered indicative of an acceptable model fit (Hu and Bentler, 1999).

## Results

### Preliminary Analyses

Descriptive statistics, correlation matrix, means and standard deviations of all major variables, as well as Pearson’s correlations among the study variables are shown in Table 1. Results revealed a positive and significant relationship between task-oriented climate, the three BPNs, autonomous motivation, and disciplined behavior. Academic performance in PE was significantly positively associated with task-oriented climate, the three BPNs, and discipline, while overall academic performance was significantly positively associated with relatedness and PE academic performance.

**TABLE 1:** Descriptive analyses, values, and correlations.

	*M*	*SD*	1	2	3	4	5	6	7
(1) Task climate	4.09	0.65							
(2) BPN autonomy	3.19	0.84	0.52**						
(3) BPN competence	3.79	0.82	0.49**	0.58**					
(4) BPN relatedness	4.18	0.84	0.42**	0.47**	0.50**				
(5) Autonomous motivation	3.82	0.92	0.67**	0.55**	0.61**	0.43**			
(6) Discipline	4.26	0.59	0.63**	0.42**	0.48**	0.50**	0.50**		
(7) PE performance	3.72	1.07	0.13*	0.12*	0.28*	0.27**	0.08	0.26**	
(8) Academic performance	3.65	1.02	0.10	−0.01	0.03	0.15**	−0.04	0.24**	0.63**

*Correlation is significant at 0.05 level; **Correlation is significant at 0.01 level. BPN, basic psychological need; PE, Physical Education.

### Structural Equation Modeling

A complete structural regression model was presented to test the study hypothesis, including antecedents (task climate), predicting BPNs (which in turn will predict motivational process), and, thereafter, the consequences (discipline, PE academic performance, and overall academic performance). The model shows adequate adjustment indexes: χ^2^ = 2464.38; *p* ≤ 0.001; χ^2^/df = 2.56, CFI = 0.90, TLI = 0.90, SRMR = 0.07, and RMSEA = 0.04. The model explained 46% of the variance in discipline, 2% in PE academic performance, and 1% in overall academic performance.

**FIGURE 1:**
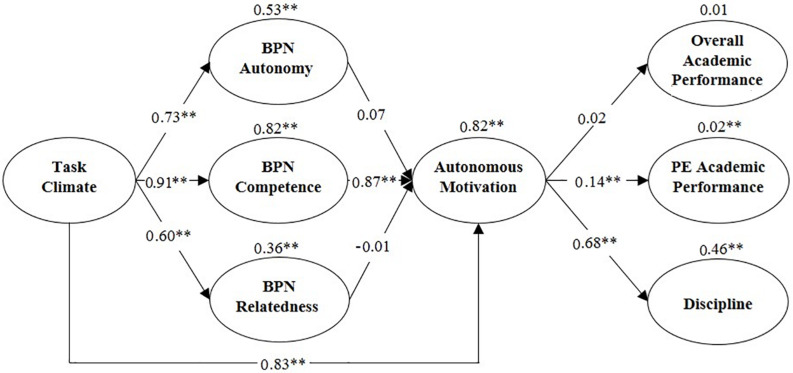
Structural equation model with standardized weights, variances and significance. ^∗^Significance level at *p* < 0.05, ^∗∗^significance level at *p* < 0.01; BPN, basic psychological need; PE, Physical Education.

Figure 1, which shows the latent variables, illustrates that the task climate has a positive effect on BPNs (autonomy β = 0.73, *p* < 0.001; competence β = 0.91, *p* < 0.001; and relatedness β = 0.60, *p* < 0.001). BPNs have a positive effect on autonomous motivation (autonomy β = 0.07, *p* = 0.77; competence β = 0.86, *p* < 0.001; relatedness β = −0.01, *p* = 0.85). Autonomous motivation has a positive effect on PE academic performance (β = 0.14, *p* < 0.001) and discipline (β = 0.68, *p* < 0.001), but with no effect on overall academic performance (β = 0.02, *p* = 0.32). Moreover, there is an indirect effect of basic task climate and autonomous motivation, mediated by the three BPNs (β = 0.83, *p* < 0.001).

## Discussion

The aim of this study was to determine a predictive model of disciplined behaviors and academic performance in PE secondary school, integrating AGT and SDT constructs. A SEM was tested in order to determine the hypothesis of the study. The results showed the relevance of task-oriented climate and the mediating role of BPNs and autonomous motivation to predict students’ disciplined behavior and PE academic performance. The study could support PE teachers’ interventions in order to achieve student adaptative consequences.

The first hypothesis, stating that *task climate will predict autonomous motivation through the basic psychological needs*, was confirmed by the results. The task-oriented climate predicted BPNs, which, in turn, predicted autonomous motivation. Our findings are consistent with previous research integrating AGT and SDT frameworks, evidencing the relationship between motivational climate and student motivation (Braithwaite et al., 2011; Duda, 2013; Cid et al., 2019). As an example, Standage et al. (2006) found a positive relationship between the motivational climate involving the task and self-determined motivation (intrinsic). In a predictive study, Gutiérrez et al. (2010) examined the relationship among pupils’ perceptions of the motivational climate, and pupils’ intrinsic motivation in PE in a sample of 2189 Spanish adolescents, aged between 13 and 17 years. They tested a SEM, and the most important predictors of pupils’ intrinsic motivation were the perceived mastery climate. In the same line, Baena-Extremera et al. (2015) and Jaakkola et al. (2017) tested other SEMs and also task-oriented climate predicted self-determined motivation. In addition, a motivational climate intervention has produced changes in students’ self−determined motivation in school PE classes (Bortoli et al., 2017). In a recent study, Cecchini et al. (2020) proposed an intervention throughout an academic year in PE to encourage a task-oriented climate, in which an increase in self-regulated forms of motivation was found.

Basic psychological needs are considered to modulate the effects of socio-contextual factors (e.g., PE teacher attitude) on the students’ self-determined motivation (Vasconcellos et al., 2019; Ryan and Deci, 2020). In a previous study involving 155 high school students, Moreno et al. (2011) stated that task-oriented climate was positively and significantly associated with the three BPNs, as in the present study. In the same line, the work developed by Kirby et al. (2015) showed that the style and the intervention of the teacher in the PE classes determined the satisfaction of the three BPNs. Another example was the intervention study conducted by Abós et al. (2017) in which the experimental group reported significant improvements on the three BPNs through an acrosport teaching unit based on developing a task-oriented motivational climate. Similarly, Bechter et al. (2019) demonstrated the positive effects of a teacher training-based program on student-centered learning intervention on BPNs in 554 Australian PE students. Following the same line, Gil-Arias et al. (2020) compared a student-centered intervention to a traditional teacher-centered one using AGT and SDT as a framework to explain how the social–contextual factors surrounding one teacher’s employment of these two pedagogical approaches influenced students’ motivation levels. They found that student-centered learning context improves the BPNs as in the present study, where the task-oriented climate improved the BPNs satisfaction of autonomy, competence, and relatedness.

However, despite the fact that many studies highlighted the role of classroom climates in catalyzing a higher student autonomous motivation and the modulating effect of BPNs on student autonomous motivation (Braithwaite et al., 2011; Duda, 2013; Vasconcellos et al., 2019), only some of them analyzed the task-oriented motivational climate as an antecedent of the motivational process in PE, including the mediating role of BPNs (Cox and Williams, 2008; Sevil et al., 2016; Abós et al., 2018). An intervention program based on task-oriented climate was applied by Sevil et al. (2016), and they found that a student-centered learning context enhances BPNs of autonomy and competence, as well as students’ intrinsic motivation. Moreover, Cox and Williams (2008) tested structural equation modeling, showing that competence, autonomy, and relatedness satisfaction mediated the relationship between teacher climate and self-determined motivation as well as in our current study. However, in the study by Cox and Williams (2008), the BPN of satisfaction significantly and positively predicted the self-determined motivation, while in the present study, only competence acted as a significant predictor of autonomous motivation. These results are also in line with those of Sevil et al. (2016) in that the BPN of competence significantly increased after an intervention program. As competence satisfaction refers to the people’s need to believe that they are effective in a specific situation context, and based on the obtained results, teachers should prioritize its support by using positive feedback, questioning and adapting the tasks to the student developmental needs and competence level (Abós et al., 2018).

In summary, the developed studies in PE (as well as in sport context) reveal the importance of the task-oriented climate and the role of BPNs in the emergence of more self-determined forms of motivation (Aelterman et al., 2013). Teacher intervention should have a strong influence on the BPNs and consequently on the students’ autonomous motivation. Thus, the PE teacher must involve students in decision-making in the process of organizing and directing the sessions, favoring their autonomy and leadership; success must be defined and evaluated in terms of effort and personal progress; mistakes should be part of the teaching–learning process; participation and commitment to the proposed activities should be emphasized; the tasks set must be varied and adjusted to the level of competence of the students; a wide range of teaching styles should be employed (cognitive, creative, socializing, individualizing, and participatory teaching styles); different strategies for grouping students, as well as a private evaluation, meaningful and relative to personal progress and mastery of the task, should be used (Moreno et al., 2011; Bortoli et al., 2017; Cecchini et al., 2020).

The second hypothesis stated that *autonomous motivation will positively predict disciplined behaviors*. The results of the study confirmed the second hypothesis. Autonomous motivation positively predicted disciplined behaviors. Our findings are in line with previous research that demonstrated the predictive capacity of self-determined motivation on prosocial behaviors (Hodge and Lonsdale, 2011; Adie et al., 2012; Sánchez-Oliva et al., 2014). Based on SDT, Sánchez-Oliva et al. (2014) tested a complete model of structural regression in PE students by analyzing antecedents, motivational process, and positive disciplined behaviors. The results showed how BPNs support predicted student autonomous motivation through the BPNs satisfaction, and the self-determined motivation predicted positive discipline behaviors in PE (respect, self-control, cooperation, and tolerance). The role of the teacher should be based on the establishment of different sets of strategies aimed at increasing the students’ confidence, being able to contribute to the teaching–learning process with a number of positive aspects. Among these aspects, the participation in the tasks by the students with an optimal level of self-esteem, the development of a proactive attitude, the promotion of the capacity for teamwork, and the perception of having less fear for making mistakes among the rest of colleagues are worth mentioning. All these aspects will lead to a greater feeling of union and belonging among the students, as well as the perception of a task-oriented motivational climate that will incite the students to have a positive predisposition toward the learning tasks, leading to disciplined behaviors (Tessier et al., 2010; Sánchez-Oliva et al., 2015).

Most of the research on disciplined behaviors has been based on AGT, finding a strong relationship between students’ perception of a motivational climate involving the task and discipline in PE (Spray, 2002; Cervelló et al., 2004; Moreno et al., 2011). The study developed by Vera and Moreno-Murcia (2016) revealed that students with motivational profiles oriented to the task showed more self-determined reasons to be disciplined. Similarly, Martínez-Galindo et al. (2012) showed that the strategies based on the teacher’s responsibility and intrinsic reasons to maintain discipline were related to the perception of a task-oriented motivational climate and with the students’ disposition to be disciplined. Task-oriented motivational climate also predicted the student identified and intrinsic reasons for being disciplined. Similarly, Moreno-Murcia et al. (2008a) found that task-oriented students perceived the strategies used by the teacher to maintain discipline in the classroom based on cooperation with others and responsibility in their own behavior, resulting in an increased self-determined motivation. The authors pointed out the importance of creating learning-oriented classroom environments because it also guided students toward a positive discipline, due to the high predictive capacity that this type of climate has shown in the tested model. Likewise, teachers must transmit implicit and explicit keys oriented toward effort, personal improvement, and acquisition of skills, which will lead to an increase in self-determined motivation. As a consequence, students will show more disciplined behavior (Adie et al., 2012).

In PE school context, disciplined behaviors have been approached from different motivational theories in an isolated manner (AGT or SDT). There are few studies that have been carried out integrating the constructs of the AGT and the SDT. We could find only one study where both constructs are integrated (Moreno et al., 2011), but they observed that the motivational climate was the main predictor of the discipline, rather than autonomous motivation in a regression analysis. This is why the present study represents an improvement when evaluating how the classroom climate predicts disciplined behaviors of the students, mediated by the autonomous motivational processes of the students in a complete predictive model. In brief, the main purpose for teachers would be to favor the appearance of disciplined behaviors through the application of interactive skills (e.g., open tasks in the form of problem situations, which encouraged personal challenge and self-regulation of learning) that allow students to perceive a task-oriented motivational climate. This perception will lead to a better achievement of BPNs and, as a consequence, a more autonomous motivation leading to disciplined behaviors.

The third hypothesis stating that *autonomous motivation will positively predict academic performance* was further confirmed by the results. Autonomous motivation positively predicted PE academic performance. Our findings are consistent with previous research based on SDT such as the longitudinal study by Barkoukis et al. (2014), which, after a 3-year intervention period, demonstrated that self-determined profiles were associated with higher ratings in the PE subject. Similar results were found by Standage et al. (2006) regarding the levels of self-determined motivation reported by students who positively predicted PE teacher qualifications. The assumption that autonomous motivation increases positive outcomes not only in the educational context (Ryan and Deci, 2020) but also in PE (Standage et al., 2006; Cid et al., 2019) is fundamental to SDT.

On the other hand, the AGT postulates that the motivational climate generated by the teacher (e.g., the keys to success or failure that define an activity) can influence positively or negatively at different levels in the classroom. In the same way, the study developed by Gutiérrez and López (2012b) found a positive and significant association between the task-oriented learning climate promoted by the teacher and the students’ grades in PE. Similar results were found by Şahin et al. (2018), showing that the task climate was determined as predictor of academic achievement among PE and sports undergraduate students. Thus, the way the teacher structures and develops his classes can generate in students a series of adaptive behaviors in the classroom, including academic performance (Sevil et al., 2017).

As previously described in the literature, academic performance has been addressed from AGT and SDT in an isolated manner in the PE context, but few studies have been carried out by integrating both frameworks or perspectives. An exception is the study developed by Sevil et al. (2017), revealing a significant and positive relationship between task-oriented climate, BPNs, self-determined motivation, and academic performance. However, the study conducted by Sevil et al. (2017) in Spanish secondary students showed that the task-oriented motivational climate (instead of the autonomous motivation) predicted academic performance in PE (35% of the explained variance). Similarly, Cerasoli et al. (2014) sought to clarify the relationships between intrinsic motivation, mastery goal orientation, and performance by using a three-wave panel study and hypotheses drawn from SDT and AGT. They reported that mastery goals mediated (explained) the relationship between intrinsic motivation and academic performance.

Despite the high correlation found in the present study between the PE grades and overall grades, autonomous motivation toward PE did not act as a significant predictor of overall academic performance. These results are not in line with previous studies in that self-determined motivation predicted academic performance in other subjects (Wentzel, 2017). A large empirically based literature has demonstrated the positive relationships of the most autonomous forms of classroom motivation with academic outcomes (Howard et al., 2017). Specifically, Taylor et al. (2014) conducted a meta-analysis highlighting the significant role of intrinsic motivation on predicting school achievement. The results may be due to the instruments used for the study, where motivation of a specific subject (PE) was analyzed, and need not be in agreement with students’ overall academic motivation. The prospective to measure motivation from a more contextual point of view is proposed, assessing the possible level of prediction of the students’ motivation on their specific and overall academic performance (Baños et al., 2020).

Traditionally, academic performance in PE has been associated with individual factors, but more and more studies indicate the teacher–student and student–student interactions as well as motivational processes as key factors of academic performance in PE (Sevil et al., 2017). In this sense, it is essential that the teacher favors task-oriented climates that satisfy BPNs, leading to autonomous motivation. Some authors point out that cognitive skills (intelligence, hours of study) are the most affected as determinants of academic achievement in the literature (Poropat, 2011), which could explain the low prediction rate of motivational processes on performance in PE. However, the study of motivational factors can provide a more complete perspective for the teaching–learning process to conclude successfully.

There are few studies that have jointly analyzed motivation, disciplined behaviors, and academic performance. Based on AGT, Gutiérrez and López (2012b) analyzed motivation, students’ behavior, and academic achievement in a sample of 2,189 PE students. The results showed that the task-oriented climate predicted the discipline behavior, although the best predictor of academic performance was the teachers’ assessment of student behavior. Complementary, Baños et al. (2018) analyzed the effect of teachers’ gender on the motivational climate, disciplined behaviors, future practice intentions, and academic performance. Finally, a recent study by Cid et al. (2019) tested a SEM where PE grades were predicted by autonomous motivation and in turn by BPNs, which were also predicted by task-oriented climate. Therefore, the motivational climate in PE classes enhanced by the PE teacher has a significant impact on the BPNs satisfaction. This fact has a positive impact on how PE students regulate their behavior, as well as on how the autonomous regulation has a significant impact in PE grades (Cid et al., 2019). The teaching role is decisive in creating the best contextual conditions to generate adaptive behaviors on students and to carry out the teaching–learning process in order to culminate in a successful manner as well.

Thus, the testing of a predictive model including disciplined behaviors and academic performance as positive student outcomes of motivational processes (integrating AGT and SDT frameworks) is the main strength of this study. The results are in line with the postulates of the Hierarchical Model of Motivation (Vallerand, 2001), which emphasizes the importance of antecedents in the emergence of more self-determined motivation forms and its consequences. In addition, this study provides a more complete knowledge of the motivational processes underlying disciplined behaviors and the academic performance of PE secondary students. The main limitation of the study is the cross-sectional design, which does not allow generalizations such as the experimental designs that are proposed as prospective. It would be also appropriate to continue to dig into this research topic but in other educational stages and including other antecedents regarding the “dark side” of student motivation as ego-oriented climate, controlled motivation, amotivation, and other consequences, such as undisciplined behavior, extracurricular sports practice, or physical activity levels. Furthermore, it would be also interesting to propose a triangulation of methods, obtaining information from the teacher and external observers. To conclude, this study contributes to the knowledge of students’ behavior and will help teachers to foment environments that optimize the teaching–learning process and lead students to a more autonomous motivation and its positive cognitive (PE academic performance) and behavioral (discipline) consequences.

## Data Availability Statement

The raw data supporting the conclusions of this article will be made available by the authors, without undue reservation.

## Ethics Statement

The University Ethics Committee of the University of Extremadura reviewed and approved the research in accordance with the principles set out in the Declaration of Helsinki. Written informed consent to participate in this study was provided by the participants’ legal guardian/next of kin.

## Author Contributions

FC and AG-A conceptualized and designed the study. FC, AG-A, LM-A, and MC recruited the subjects. MC and LM-A collected the data. FC and MC organized the database. FC and AG-A carried out the statistical analysis. FC, LM-A, MC, and AG-A wrote the first manuscript draft. FC, AG-A, and LM-A developed the final manuscript draft, contributed to English proofreading, and reviewed and edited the final version of the manuscript. All authors contributed to manuscript revision and approved the definitive manuscript.

## Conflict of Interest

The authors declare that the research was conducted in the absence of any commercial or financial relationships that could be construed as a potential conflict of interest.
